# EVALUATION OF TREATMENT PARAMETERS FOR FOCUSED-EXTRACORPOREAL SHOCK WAVE THERAPY IN KNEE OSTEOARTHRITIS PATIENTS WITH BONE MARROW LESIONS: A PILOT STUDY

**DOI:** 10.2340/jrm.v56.13207

**Published:** 2024-03-12

**Authors:** Hani AL-ABBAD, Jacqueline E. REZNIK, Erik BIROS, Bruce PAULIK, Rob WILL, Samuel GANE, Penny MOSS, Anthony WRIGHT

**Affiliations:** 1 Physical Therapy Department, Rehabilitation Hospital, King Fahad Medical City. Riyadh, Saudi Arabia; 2 College of Healthcare Science and Division of Tropical Health and Medicine, James Cook University, Townsville, QLD, Australia; 3 College of Medicine and Dentistry, James Cook University, Townsville, Australia; 4 Hollywood Functional Rehabilitation Clinic, Nedlands; 5 School of Medicine and Pharmacology, University of Western Australia, Perth; 6 SKG Radiology, Perth; 7 School of Allied Health, Curtin University, Perth, WA, Australia

**Keywords:** bone marrow lesions, extracorporeal shock wave therapy, knee osteoarthritis, single-case experimental design

## Abstract

**Objectives:**

To evaluate the effect of different dosage parameters of focused-extracorporeal shock wave therapy on pain and physical function in knee osteoarthritis patients with bone marrow lesions. In addition, to investigate pathophysiological changes based on imaging and biomarker measures.

**Methods:**

Using a single-case experimental design, a total of 12 participants were randomly allocated in 4 equal groups of 3 to receive different dosages of focused-extracorporeal shock wave therapy. Each group received either 4 or 6 sessions of 1500 or 3000 shocks over 4 or 6 weekly sessions. Participants underwent repeated measurements during the baseline, intervention, and post-intervention phases for Western Ontario and McMaster Universities Osteoarthritis Index (WOMAC) score, aggregated locomotor function score and pressure pain threshold. Imaging and inflammatory biomarker outcomes were measured at baseline and 3 months following the intervention.

**Results:**

The group receiving the highest dosage of focused-extracorporeal shock wave therapy showed clinical improvements superior to those of participants in the other 3 groups. Statistically significant changes during the follow-up phase in contrast to baseline measurements for the WOMAC score (Tau-U= –0.88, *p* < 0.001), aggregated locomotor function score (Tau-U= –0.77, *p* = 0.002), and pressure pain threshold (Tau-U= 0.54, *p* = 0.03) were observed. Bone marrow lesion and inflammatory cytokines demonstrated no change.

**Conclusion:**

A dose-dependent effect for focused-extracorporeal shock wave therapy on osteoarthritis-related symptoms was suggested. However, these improvements were not associated with changes in the underlying pathophysiological mechanisms.

LAY ABSTRACT

Knee osteoarthritis is a well-described chronic low-grade inflammatory joint disease that affects all components of the joints. Bone marrow lesions are well-defined knee osteoarthritis markers that could accelerate the disease progression. Extracorporeal shock wave therapy is becoming a popular treatment choice for degenerative joint conditions; however, the optimal dosage parameters are not clear. In this study, 4 different treatment dosages were evaluated in patient with painful knee osteoarthritis and imaging evidence of bone marrow lesion. This study found that the group of patients receiving the highest dosage parameters showed superior self-reported and measured clinical outcomes. However, these improvements were not associated with changes in the underlying pathological state of the disease.

Knee osteoarthritis (OA) is one of the most common disabling forms of arthritis, leading to reduced quality of life, dependency, and economic burden to health systems and patients (1). Loss of articular cartilage and osteophyte formation is the fundamental underlying pathology of osteoarthritis (2). However, it is also essential to consider OA as a whole joint disease with multiple OA phenotypes based on distinct characteristics such as structural changes detectable by imaging, biochemical features, and clinical presentation (3). Subchondral bone deterioration and sclerosis are crucial components of the disease and are currently considered strong early predictors for late articular cartilage damage (4).

Subchondral bone marrow lesions (BML) are a common feature seen in knee OA, which are identified as poorly defined hyper-intense signals on magnetic resonance imaging (MRI). They involve a histopathological combination of necrosis and fibrosis, bone marrow oedema, cysts, microfractures with bleeding, fibrovascular ingrowth and remodelled trabeculae (5). The pathophysiological basis for BML-related pain is not fully explained. However, suggested multifactorial mechanisms include direct insult and increased intramedullary pressure causing irritation to neurovascular structures and venous drainage impairment, resulting in local acidosis and secretion of inflammatory cytokines (6, 7). Early detection and treatment of BML in individuals with knee OA are therefore essential for both clinical improvement and prevention of further disease progression.

Among conservative non-surgical therapies, extracorporeal shock wave therapy (ESWT) is considered the treatment of choice for different recalcitrant musculoskeletal conditions including knee OA. The focused- ESWT type is a non-invasive system generating high-energy pressure waves through electrohydraulic, electromagnetic, or piezoelectric mechanisms (8). It is believed that ESWT effects on targeted tissues are due to the mechanotransduction process whereby the mechanical energy of the pressure waves can cause subsequent biological effects in both intracellular and extracellular structures. These processes include cellular metabolic changes that result in tissue regeneration and healing (9).

A few studies have investigated the effects of focused-ESWT on knee OA with BML, reporting improvement in both clinical and radiological outcomes (10–13). These studies used high-dosage parameters for focused-ESWT. However, this high dose of focused-ESWT has also been reported to result in pain and local tenderness that may require local anaesthesia leading to lower ESWT effectiveness (14). It is unknown if a lower intensity focused-ESWT dose will produce similar effects on knee OA to a high intensity treatment in humans, with less pain.

Therefore, the primary aim of this study was to investigate the effects of different focused-ESWT dosage parameters on the pain and function of individuals with symptomatic knee OA and BML. The secondary aim was to evaluate whether the application of focused-ESWT is associated with changes in imaging of subchondral BML, bone mineral density (BMD), and changes in circulating biomarkers of inflammation.

## METHODS

The study protocol was approved by Curtin University Human Research Ethics Office (HRE2019-0219).

### Study design

The study design was a prospective single-case experimental A-B-A design (SCED). In particular, the participants underwent repeated measurements at baseline (A), during the intervention (B), and post-intervention (A) periods for all pain and functional outcomes. Four baseline measurements were taken at 1-week intervals for all participants. During the intervention phase, 4 or 6 weekly measurements were undertaken based on the allocation of the participants into groups. Four post-intervention follow-up measurements were carried out at 3-week intervals for all participants. MRI of the affected knee and bilateral knee dual-energy X-ray absorptiometry (DXA) scans were conducted for all participants at baseline and 3 months following the last treatment. In addition, blood samples were collected from all participants at baseline, end of treatment, and 3 months following the last treatment visit to measure inflammatory biomarkers. The study was conducted at a physiotherapy practice in Perth, Western Australia.

### Participants

Recruitment of participants took place between September 2019 and June 2020 through a advertisement on Curtin Radio , which broadcasts across all suburbs in Perth, Western Australia. Individuals with diagnosed knee OA were screened for suitability by a consultant rheumatologist according to the American College of Rheumatology (ACR) clinical classification criteria for knee OA (15). Eligibility criteria included evidence of bone marrow lesion (BML) according to MRI with a Whole Organ Magnetic Imaging Score (WORMS) of ≥1 at any subregion. Exclusion criteria included: history of neurological disorders affecting sensory, motor, or cognitive function; recent lower limb injury or surgery; previous treatment with any form of ESWT or intra-articular injection into the affected knee during the last 6 months. Eligible participants were provided with detailed information about their involvement in the study and were required to provide written informed consent.

### Intervention

Focused-ESWT was administered using the ORTHOSPEC COMPACT system by Medispec Int. (Gaithersburg, MD 20877, USA) powered by an electrohydraulic method of shock-wave generation. Computer-generated random allocation sequences allocated 12 participants to 4 focused-ESWT groups: group A received 3000 shocks over 4 sessions, group B received 3000 shocks over 6 sessions, group C received 1500 shocks over 4 sessions, and group D received 1500 shocks over 6 sessions. According to the MRI findings and the participant’s pain response, the application area was adjusted and focused based on the BML anatomical location. The intervention was delivered by an experienced physiotherapist, accredited in ESWT clinical application. The focused-ESWT dose parameters were determined in conjunction with a recommendation from the device manufacturer. Each participant’s energy level was adjusted based on their pain response and could be modified within and between treatment sessions to maintain a treatment intensity that was strong but tolerable for each individual.

### Outcome measures

*Pain and functional outcomes.* The Western Ontario and McMaster Universities Osteoarthritis Index (WOMAC, VA3.1) measures self-reported pain, stiffness, and daily activities restriction. The WOMAC is a commonly used tool to quantify knee OA-related pain and disability that demonstrates good internal validity and test-retest reliability (16). The WOMAC has 24 items each scored on a 100-mm visual analogue scale (VAS) with a maximum possible score of 2400 points, indicating self-reported worsening across the 3 domains.

Physical function was measured using the 3 tasks of the aggregated locomotor function (ALF) test in standardized order (17). Time taken to complete the 3 tasks was measured: sit-to-stand (stand from a chair, walk 2-m, return and sit again); walk (8-m); stairs (10-step ascent and descent). Each task was repeated 3 times, and the mean time in seconds was recorded for analysis. This score has shown excellent inter-rater reliability and good responsiveness following interventions (17).

Pressure pain threshold (PPT) was measured using an electronic digital pressure algometer (Somedic AB, Sösdala, Sweden), a device that shows good reliability when applied by a skilled assessor (18). The area of maximum tenderness over the medial tibial plateau of both knees was identified through palpation and marked. The mean of 3 measurements was used for analysis. The test was performed in the side-lying position using a 1-cm^2^ probe applied at 40 kPa/s, placed perpendicular at 90^o^ knee flexion. Participants were asked to press a button at the point when the initial sensation of pressure became painful (19).

The Global Rating of Change (GRoC) was assessed using an 11-point scale (from –5, very much worse to +5, completely recovered with 0 indicating unchanged) at the final follow-up visit at 3-month post-treatment (20). Using this scale, participants were asked to describe themselves now compared with before receiving the focused-ESWT with respect to the knee osteoarthritis. The advantage of using this scale is to allow participants to provide evaluation of their overall condition following the treatment received.

Participants were advised to report any adverse events either during or following the focused-ESWT treatment sessions. All participants were advised at the start that the focused-ESWT may cause pain or discomfort during the treatment session and were asked to report any pain >4/10 on a visual analogue scale (VAS).

*Imaging outcomes.* Semi-quantitative assessment of BML using the WORMS (21) was performed. BML was assessed on a 1.5 T magnetic resonance imaging (MRI) unit (Siemens, Germany) with the following protocol: T2-weighted fat-suppressed fast spin-echo, sagittal images obtained at a slice thickness of 2.2–3 mm, slice gap=0.2–0.4 mm, and coronal images of 2.5–3 mm slices with 0.3–0.6 mm gap. The WORMS has 15 subregion divisions of the knee: patella (medial, lateral), medial/lateral aspects of femur and tibia (anterior, central, posterior), and the sub-spinous tibia region. Total BML score is the sum for each subregion from 0 to 3 according to the percentage of subregional BML volume (0=none; 1 ≤25%; 2=25–50%; 3 ≥50% of the region). The 2 patella subregions were not scored for this study as the focused-ESWT intervention was not targeting the patella bone. The WORMS scoring of the knee BML has good reported inter-reader reliability (22). All MRIs were scored independently by the same experienced and blinded musculoskeletal radiologist at baseline and 3 months following focused-ESWT using the InteleViewer™ software (Intelerad, version 4-18-1-P204).

Assessment of knee subchondral BMD was performed using DXA (Lunar prodigy) by a licensed radiology technician based on a semi-standardized protocol (23, 24). The proximal tibia was used as the Region of Interest (ROI). The acquisition view was an anterior/posterior view with the patient supine and the knee in extension with the hip internally rotated. The tibial ROI was defined manually as the tibial cortical surface forming the upper border, and the lower border extending 10 mm distally, with the width extending from the medial tibial border to the medial joint edge. It only included the loading zone of the tibia subchondral trabecular bone. The lateral tibial plateau was measured in a similar manner using contralateral direction and landmarks. Both knees were assessed independently at baseline and 3 months after the focused-ESWT intervention. Intra-rater reliability was tested using a 2-way random effect model with excellent agreement (intraclass correlation coefficient (ICC)= 0.91, 95% confidence interval (95% CI) 0.84, 0.95, *p*<0.001).

*Biomarker outcomes.* Blood samples were collected in 5-mL serum gel separator tubes from an antecubital vein with the participant in the sitting position. The blood samples were allowed to clot for 20–30 min before centrifuging at room temperature. Samples were then centrifuged for 15 min at 1400 rounds per second (RPS), and the supernatant was separated as serum using disposable transfer pipettes and stored at –75°C until analysed. Sandwich enzyme-linked immunosorbent assays (ELISAs) were used to quantify the serum concentrations of inflammatory biomarkers using the following commercial ELISA kits: tumour necrosis factor α (TNF-α ABTS ELISA, PeproTech, Cranbury, NJ, USA), C-reactive protein (CRP Quantikine ELISA kit (R&D Systems Inc., Minneapolis, MN, USA) and interleukin 1β (IL-1β), Interleukin 6 (IL-6) (Duoset, R&D Systems Inc., Minneapolis, MN, USA) according to the manufacturer’s instructions.

### Statistical analysis

Data were analysed using SPSS for Windows (version 26, IBM Corp, Armonk, New York, USA). All pain and functional outcomes data (WOMAC, ALF, and PPT) for each focused-ESWT group were plotted for trend visual inspection within baseline and between phases. Three features of visual inspection can suggest a non-overlap relation between the measured phases. These features include the central tendency (the mean) for data within each phase, estimating a trend based on the slope of the best fitted straight line, and estimating the variability of data around the best-fitted slope (25, 26). The effect size estimates associated with the application of different focused-ESWT parameters were calculated using the Tau-U method. The Tau-U index is preferred for measuring non-overlap data between 2 phases using a non-parametric technique (27). The Tau-U Calculator (http://www.singlecaseresearch.org/calculators/tau-u, Single Case Research, USA) was used to analyse all Tau-U indices. Baseline correction was performed when a positive trend was observed.

According to data distribution, descriptive statistics are expressed as means with standard deviations (SD) or medians with interquartile ranges (IQR). The change from baseline to post-intervention and follow-up was analysed using Friedman’s related-samples 2-way analysis of variance (ANOVA) for the inflammatory biomarkers. The selection of the non-parametric statistic is to account for the heterogeneity of variances related to the variables. In addition, a paired-samples *t*-test was used to examine the change from baseline to follow-up for the WORMS and BMD. The significance level was assumed at *p*<0.05.

## RESULTS

### Participants’ characteristics

Twelve participants (8 females) with knee OA and BML were recruited and randomly allocated 3 per group, making 4 groups with different doses of focused-ESWT, as detailed above. The mean (SD) age was 70.17 (5.37) years, and mean (SD) body mass index (BMI) was 28.54 (4.89) kg/m^2^. The between-group differences at baseline for age, BMI, WORMS and BMD were not significant (p > 0.05). All participants completed the follow-up phase. Descriptive characteristics are shown in Table I according to the treatment group allocation.

**Table I:** Participants’ baseline characteristics

Variable	Group A	Group B	Group C	Group D	*p*-value
N	3	3	3	3	
Sex (male/female)	1/2	2/1	1/2	0/3	
Age (years)	74.33 (6.02)	72 (4.58)	67.33 (6.03)	67 (2.65)	0.277
BMI (kg/m^2^)	26.95 (1.84)	27.10 (3.68)	30.81 (7.81)	26.9 (6.18)	0.788
Symptom onset (months)*	36 (114)	24 (24)	18 (54)	60 (42)	0.743
Affected knee (R/L)	0/3	1/2	1/2	2/1	
Number of focused-ESWT shocks per session	3000	3000	1500	1500	
Number of sessions	4	6	4	6	
Total number of shocks	12000	18000	6000	9000	

* Median (interquartile range). ESWT: extracorporeal shock wave therapy; BMI: body mass index.

### Effects on pain and functional outcomes

*Western Ontario and McMaster Universities Osteoarthritis index.* A statistically significant change was shown in groups B (3000 shocks over 6 sessions) and D (1500 shocks over 6 sessions) from baseline to the intervention phase (Tau-U= –0.63, *p* = 0.006; –0.72, *p* = 0.002). A similar change was shown during the follow-up compared with baseline phase (–0.88, *p* < 0.001; –0.74, *p* = 0.004). However, group A (3000 shocks over 4 sessions) showed a worsening trend from baseline to the intervention phase (0.50, *p* = 0.046) and group C (1500 shocks over 4 sessions) from the intervention to follow-up phase (0.62, *p* = 0.016) (Table II).

**Table II:** Effect of focused-extracorporeal shock wave therapy on pain and function measures

Variable	Group A	Group B	Group C	Group D
Tau-U weighted mean (*p* value) – baseline to intervention				
WOMAC ALF PPT	0.50 (0.046)* –0.38 (0.13) 0.04 (0.87)	–0.63 (0.006)* –0.53 (0.019)* 0.69 (0.02)*	–0.43 (0.107) –0.62 (0.016)* 0.14 (0.58)	–0.72 (0.002)* –0.799 (0.001)* 0.07 (0.752)
Tau-U weighted mean (*p* value) – intervention to follow-up				
WOMAC ALF PPT	–0.58 (0.02)* 0.08 (0.74) 0.00 (1.00)	–0.83 (0.000)* –0.72 (0.001)* 0.028 (0.90)	0.62 (0.016)* 0.03 (0.91) 0.23 (0.36)	–0.25 (0.27) –0.03 (0.90) 0.03 (0.90)
Tau-U weighted mean (*p* value) – baseline to follow-up				
WOMAC ALF PPT	–0.13 (0.62) –0.71 (0.005)* –0.08 (0.73)	–0.88 (0.000)* –0.77 (0.002)* 0.54 (0.03)*	0.22 (0.385) –0.25 (0.317) 0.33 (0.18)	–0.74 (0.004)* –0.91 (0.000)* –0.01 (0.97)

* Denote statistical significance (*p*<0.05).

WOMAC: Western Ontario and McMaster Universities Osteoarthritis index; ALF: aggregated locomotor function; PPT: Pressure pain threshold.

**Fig. 1:**
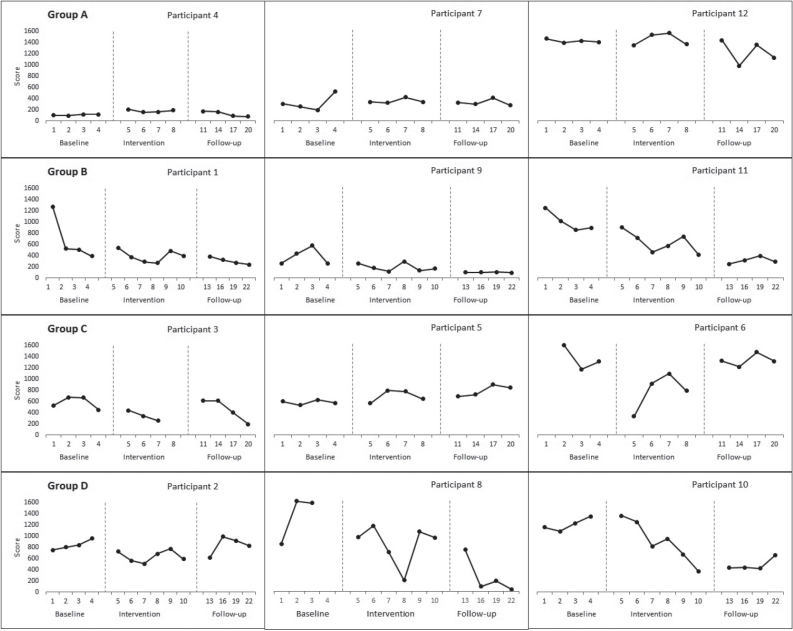
Effect of focused-extracorporeal shock wave therapy (ESWT) on Western Ontario and McMaster Universities Osteoarthritis Index (WOMAC) score.

A therapeutic reduction trend was observed on the individual visual inspection in 2 participants of groups B (3000 shocks over 6 sessions) and D (1500 shocks over 6 sessions) from baseline to the follow-up phase. Group A (3000 shocks over 4 sessions) and C (1500 shocks over 4 sessions) individual data had high variability, indicating a non-conclusive trend except for 2 participants in group C (1500 shocks over 4 sessions) who improved during the intervention phase compared with their respective baseline measurements (Fig. 1).

**Fig. 2:**
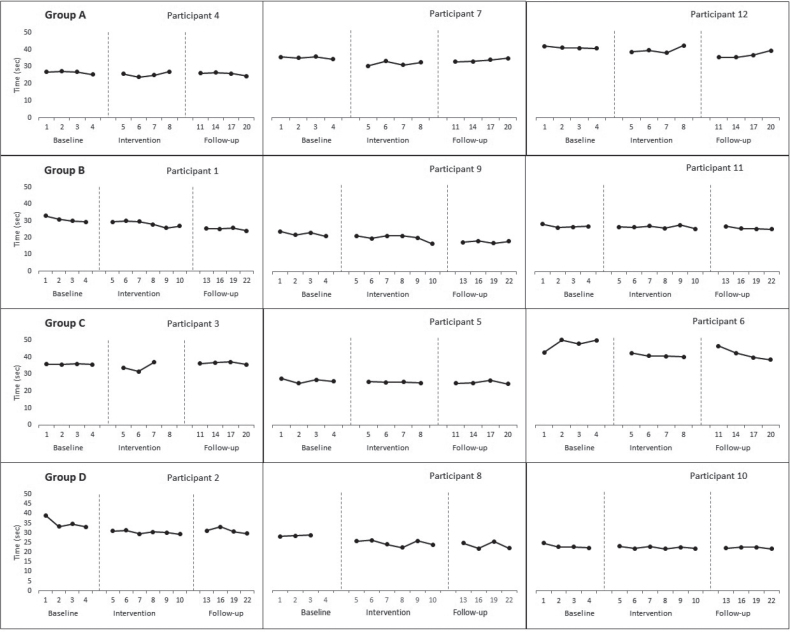
Effect of focused-extracorporeal shock wave therapy (ESWT) on aggregated locomotor function (ALF) score.

*Aggregated locomotor function score.* The ALF score improved across all groups except group A (3000 shocks over 4 sessions) from baseline to intervention phase (Group B= –0.53, *p*=0.02; Group C= –0.62, *p*=0.02; Group D= –0.80, *p*=0.001). A significant change between the intervention and follow-up phases was shown only in group B (3000 shocks over 6 sessions) (–0.712, *p* = 0.001). Apart from group C (1500 shocks over 4 sessions), all groups showed improvements from baseline to the follow-up phase (Group A Tau-U= –0.71, *p* = 0.005; Group B= –0.77, *p* = 0.002; Group D= –0.91 *p* < 0.001) (Table II). Visual data inspection suggested a therapeutic trend for 1 participant in group A (3000 shocks over 4 sessions) and group C (1500 shocks over 4 sessions), 2 participants in groups B (3000 shocks over 6 sessions) and D (1500 shocks over 6 sessions) (Fig. 2).

**Fig. 3:**
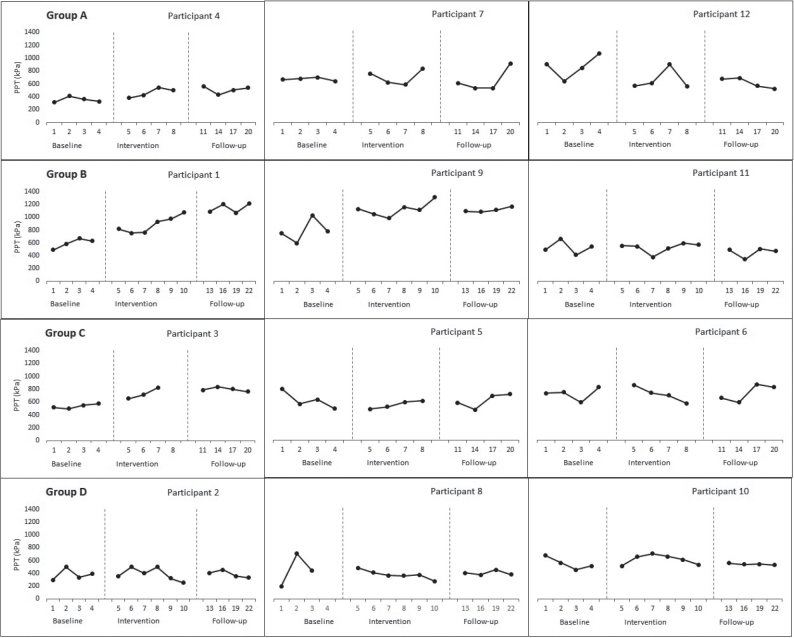
Effect of focused-extracorporeal shock wave therapy (ESWT) on pressure pain threshold (PPT).

*Pressure pain threshold.* The Tau U score revealed a statistically significant therapeutic change in PPT only in group B (3000 shocks over 6 sessions) from baseline to intervention and follow-up phases (Tau-U= 0.69, *p* = 0.02; 0.54 *p* = 0.03), respectively (Table II). However, there was no significant contrast during the follow-up compared with the intervention phase. The individual visual inspection showed that 2 out of 3 participants improved at the follow-up phase compared with baseline in group B, compared with 1 participant in both groups A (3000 shocks over 4 sessions) and C (1500 shocks over 4 sessions), but none in group D (1500 shocks over 6 sessions) (Fig. 3).

*Global rating of change score.* The GRoC score revealed an overall perception of improvement among participants regarding their treated knee condition following focused-ESWT (1.92 ± 1.93). Group B (3000 shocks over 6 sessions) scored the highest perceived improvement (3.33 ± 1.15), while group C (1500 shocks over 4 sessions) scored the lowest perceived improvement (0.3 ± 2.5). However, the between-group difference was not statistically significant (*p* = 0.33).

### Adverse events

All participants were advised at the start that the focused-ESWT may cause pain or discomfort during the treatment session and were asked to report any pain > 4/10 on a VAS. One participant reported 2 occasions of temporary tenderness (pain > 4/10) during the focused-ESWT applications. Therefore, the energy level was adjusted to minimize pain at or below 4/10.

### Effects on imaging outcomes

*Bone marrow lesion.* Using the WORMS measure, the medial tibiofemoral subregi-on (MTFS) scored higher than the lateral tibiofemoral subregion (LTFS), mean 4.75 (3.44), 2.25 (2.3); respectively at baseline. There was a modest overall reduction in the total, MTFS and LTFS WORMS following the focused-ESWT at the 3-month follow-up, but it was not statistically significant (*p* > 0.05). Furthermore, the within- and between-groups differences were not statistically significant (*p* > 0.05) (Table III).

**Table III:** Effects of focused-extracorporeal shock wave therapy on bone marrow lesion and bone mineral density

Variable	Group A Mean (SD)	Group B Mean (SD)	Group C Mean (SD)	Group D Mean (SD)	Overall Mean (SD)	*p-*value^#^
WORMS Total Baseline Total Follow-up MTFS Baseline MTFS Follow-up LTFS Baseline LTFS Follow-up	10.67 (3.79) 9 (2.65) 7 (4.58) 6 (1.73) 2.67 (2.082) 2 (1.53)	5.67 (2.31) 5.67 (3.21) 4 (2) 3.67 (1.53) 1.33 (0.58) 1.33 (1.43)	6.33 (5.51) 6.67 (5.51) 5.33 (4.51) 5 (4.58) 0.33 (0.58) 1 (1)	8.67 (6.35) 6.67 (4.73) 2.67 (2.08) 2.33 (2.08) 4.67 (2.89) 3.67 (2.89)	7.83 (4.55) 7 (3.79) 4.75 (3.44) 4.25 (2.77) 2.25 (2.3) 2 (2)	0.137 0.256 0.389
Medial tibial compartment BMD (g/cm^2^) Baseline Follow-up	1.384 (0.264) 1.261 (0.423)	1.179 (0.393) 1.103 (0.614)	1.221 (0.489) 1.42 (0.168)	1.112 (0.208) 1.044 (0.259)	1.224 (0.321) 1.207 (0.277)	0.811
Lateral tibial compartment BMD (g/cm^2^) Baseline Follow-up	1.128 (0.075) 0.976 (0.176)	0.827 (0.244) 0.8 (0.037)	0.825 (0.231) 1.002 (0.151)	1.074 (0.203) 0.994 (0.06)	0.964 (0.211) 0.943 (0.135)	0.780

# Statistical significance set at *p*<0.05.

SD: standard deviation; MTFS: medial tibiofemoral subregion; LTFS: lateral tibiofemoral subregion; WORMS: Whole-Organ Magnetic Imaging Score; BMD: bone mineral density.

*Subchondral bone mineral density.* At baseline, the mean medial and lateral tibial compartment BMD was 1.224 (0.321) and 0.964 (0.211) g/cm^2^, respectively. There was no significant within- and between-groups mean difference in the medial and lateral tibial compartment BMD following focused-ESWT (*p* > 0.05). Moreover, the mean difference was not significant between treated and non-treated knees (*p* > 0.05) (Table III).

### Effects on biomarkers outcomes

There was no significant difference in overall measured serum concentration of pro-inflammatory cytokines including C-reactive protein (CRP), tumour necrosis factor alpha (TNF-α), interleukin (IL)-1β, and IL-6, from baseline to end of treatment or at 3-month follow-up (*p*>0.05). Furthermore, there was no significant difference between treatment groups across all endpoints (Table IV). CRP overall expression showed a modest increase at the 3 months follow-up. Conversely, IL-6 overall expression showed a modest decrease at the 3-month follow-up. However, the overall expression of both TNF-α and IL-1β were close to the not detectable range at all endpoints.

**Table IV:** Effects of focused-extracorporeal shock wave therapy on inflammatory marker in people with knee osteoarthritis and bone mineral density

Variable	Group A Median (IQR)	Group B	Group C	Group D	Overall	*p*-value
CRP concentration (μg/mL) Baseline Post-intervention Follow-up	3.931 (3.491) 5.190 (14.218) 4.410 (14.019)	1.125 (1.761) 2.595 (2.810) 8.213 (27.617)	2.850 (3.022) 3.136 (1.735) 5.684 (6.376)	5.341 (8.674) 3.667 (24.765) 3.239 (2.994)	3.039 (3.805) 3.425 (2.922) 4.736 (6.471)	0.2
TNF-α concentration (ng/mL) Baseline Post-intervention Follow-up	0 (4.211) 0 (3.633) 0 (2.176)	2 (0.058) 0 (0.028) 0 (0.023)	0 (0.012) 0.017 (0.035) 0 (0.006)	0 (0.010) 0 (0.040) 0 (0.000)	0 (0.011) 0 (0.035) 0 (0.006)	0.09*
IL-1β concentration (ug/mL) Baseline Post-intervention Follow-up	0.001 (0.300) 0.003 (0.011) 0.020 (0.055)	0.013 (0.584) 0.026 (0.043) 0.019 (0.040)	0.023 (0.037) 0.017 (0.003) 0.020 (0.012)	0.538 (0.757) 0.570 (0.795) 0.447 (0.421)	0.026 (0.178) 0.018 (0.217) 0.020 (0.046)	0.4
IL-6 concentration (pg/mL) Baseline Post-intervention Follow-up	2.270 (11.548) 1.928 (21.822) 2.050 (10.822)	1.820 (2.337) 1.945 (2.163) 1.858 (2.164)	1.694 (12.635) 5.350 (7.048) 1.961 (10.440)	5.124 (13.020) 8.306 (11.408) 4.039 (3.204)	2.303 (7.489) 2.163 (7.048) 2.050 (3.782)	1.0

* Denote statistical significance (*p*<0.05).

IQR: interquartile range; CRP: C-reactive protein; TNF-α: tumour necrosis factor; IL-1β: interleukin 1; IL-6: interleukin 6.

## DISCUSSION

This pilot study evaluated whether focused-ESWT shows a dose-dependent effect in people with knee OA and BML. Furthermore, the study aimed to examine if the application of focused-ESWT influences the pathophysiological characteristics of knee OA and BML as measured by imaging and circulating biomarkers. The study’s findings suggest a dose-dependent response on all related clinical outcomes of pain and function. The participants in group B received the highest focused-ESWT dose of 3000 shocks for 6 weekly sessions and showed consistent improvement in all measured clinical outcomes. In addition, the Tau-U score provided statistical evidence of within-subject favourable improvements in WOMAC score, ALF score and PPT from baseline to intervention and follow-up phases. Likewise, the individual visual inspection of group B revealed a therapeutic trend in all participants during the follow-up phase compared with baseline for both the WOMAC and ALF scales. Similarly, the PPT visual inspection depicted a therapeutic trend in 2 out of the 3 participants at the follow-up phase compared with baseline.

These findings agree with previous research that used high-energy focused-ESWT on knee OA and BML (10–13). These previous studies used a varying range of 2000–4000 shocks per session over 2–3 sessions at either level 3 or 4 with a high energy flux density (EFD) of 0.22–0.55 mJ/mm^2^. Moreover, the results are similar to the application of radial type ESWT on people with symptomatic knee OA, where high radial ESWT intensity was more effective (28) compared with low intensity (29).

In the current study the number of shocks was standardized to 1500 or 3000 over 4 or 6 sessions and the intensity level was customized according to the patient’s perceived pain level. These treatment parameters were selected based on the device manufacturer’s guidelines, the number of shocks used in previous studies (30), and standard clinical practice. The selection of focused-ESWT dosage parameters specifically for knee OA is not well established. A recent systematic review (30) reported that the mean used EFD was 0.2 (0.09) with a range of 0.05–0.4 mJ/mm^2^, mean number of shocks 2846.9 (1812.24) and a range of 200–10,000 and the mean number of sessions was 5.8 (3.96) with a range of 3–24. Previous clinical studies determined the focused-ESWT parameters primarily based on animal studies and manufacturer’s recommendations.

The current study’s dose-dependent findings on pain and function are consistent with Liao et al.’s (30) recent systematic review and meta-analysis that evaluated the clinical efficacy of ESWT for knee OA (30). The authors provided evidence that the ESWT energy output is an independent predictor for improvement in pain. Also, the intervention period predicted improvement in function based on the WOMAC score. The current study showed that participants in the group receiving the highest dose reported superior pain and functional changes. However, despite the clinical improvements in functional pain outcomes, the current results did not provide evidence to suggest that focused-ESWT altered the morphological features of knee OA and subchondral BML.

This study utilized the WORMS tool as a reliable semi-quantitative measure for assessing BML on MRI. The findings did not support previous studies that reported either reduction or complete regression of the BML following focused shock wave treatment. Different methodological aspects may explain the variation of the current results compared with previous studies. First, we must acknowledge the small sample size used in this study, as the main objective was to evaluate different focused-ESWT dosage parameters using the single-case experimental design. Secondly, the current cohort consisted of participants diagnosed with knee OA with a mean age of 70 years and a mean duration of symptoms of over 6 months. All previous studies included much younger participants with a mean age between 40 and 60 years and a shorter symptom duration of 3–6 weeks (10–13).

Moreover, previous studies either used a quantitative measurement method of assessing the BML size (12, 13) or a categorical classification of unchanged, reduced, or completely regressed method (10, 11). Therefore, it is observed that previous studies reported a relatively large overall BML size at baseline in comparison with our relatively small baseline BML measurement. This observation could be a potential mediating factor, as a recent systematic review on the effects of shock wave on musculoskeletal conditions based on changes in imaging demonstrated that baseline lesion size was a predicting factor for the shock-wave effect (31). In addition, knee OA is a well-described chronic slow inflammatory disease, and changes in the pathological features may require longer follow-up intervals to observe change (minimum of 6 months) compared with the current study’s 3-month follow-up interval.

Similarly, subchondral BMD showed no alteration after the focused-ESWT application in both medial and lateral tibial compartments at the 3-month follow-up. The change in BMD was not different compared with the untreated knee. There are no previous studies examining the effect of shock wave treatment on knee subchondral BMD in humans. However, other studies evaluating BMD change post shock wave on other musculoskeletal conditions reported controversial results. Gerdesmeyer et al. (32) reported a statistically significant change in BMD of the treated calcaneus using focused-ESWT for 2 sessions of 2000 shocks and 0.32 mJ/mm^2^ EFD at 3 months follow-ups, providing evidence of an osteogenic effect. Likewise, a study by Shi et al. (33) reported that a high-energy focused-ESWT (0.28 mJ/mm^2^ EFD) treatment improved hip BMD in postmenopausal osteoporotic patients compared with low-energy (0.15 mJ/mm^2^ EFD) and control at 1-year follow-up. On the contrary, 1 session of unfocussed-ESWT with 3000 shocks and 0.3 mJ/mm^2^ EFD on the distal forearm of 12 postmenopausal female subjects, free of bone disease, demonstrated no effect on BMD (34).

Changes in serum pro-inflammatory cytokine concentration measured in this study (TNF-α, CRP, IL-1β, and IL-6) were not evident following the focused-ESWT. Although the concentration of both TNF-α and IL-1β were mostly non-detectable across all time-points; however, CRP, an indicator of inflammation produced by the liver, was relatively high. These findings are similar to those of Eftekharsadat et al. (35), who reported no change in serum-CRP following ESWT among patients with moderate knee OA. Likewise, a study by Brenner et al. (36) reported non-detectable levels of TNF-α in both synovial fluid and synovial membrane in people with knee OA.

The 12 participants recruited for this study were diagnosed as having knee OA via clinical assessment including X-ray and conformed to age, sex and BMI values commonly found in OA studies. However, the study did not retain X-rays and classify participants according to radiological stage of OA according to the Kellgren–Lawrence method since the focus of this pilot study was on investigation of effects of dose parameters on DXA and MRI outcomes. A future larger study would include the more standard Kellgren-Lawrence classification.

In conclusion, this single-case experimental design pilot study found improvements in pain and physical disability in patients with knee OA following focused-ESWT treatment. The improvement was dose-dependent, with more shocks and a more prolonged focused-ESWT treatment phase associated with improved OA symptoms. Since no difference was found regarding circulating biomarkers of inflammation, focused-ESWT has proven effective even within this pro-osteoarthritic inflammatory environment. Understanding the importance of treatment dosage provides useful information to support the design of future randomized clinical trials to evaluate focused-ESWT in the treatment of knee OA.
